# Rational Design of a Thermostable 2′-Deoxyribosyltransferase for Nelarabine Production by Prediction of Disulfide Bond Engineering Sites

**DOI:** 10.3390/ijms231911806

**Published:** 2022-10-05

**Authors:** Guillermo Cruz, Javier Acosta, Jose Miguel Mancheño, Jon Del Arco, Jesús Fernández-Lucas

**Affiliations:** 1 Applied Biotechnology Group, Universidad Europea de Madrid, Urbanización El Bosque, Calle Tajo, s/n, 28670 Villaviciosa de Odón (Madrid), Spain; 2 Department of Crystallography and Structural Biology, Institute Rocasolano (CSIC), Serrano 119, 28006 Madrid, Spain

**Keywords:** biocatalysis, 2′-deoxyribosyltransferase, thermal stability, structural bioinformatics, nucleoside analogues

## Abstract

One of the major drawbacks of the industrial implementation of enzymatic processes is the low operational stability of the enzymes under tough industrial conditions. In this respect, the use of thermostable enzymes in the industry is gaining ground during the last decades. Herein, we report a structure-guided approach for the development of novel and thermostable 2′-deoxyribosyltransferases (NDTs) based on the computational design of disulfide bonds on hot spot positions. To this end, a small library of NDT variants from *Lactobacillus delbrueckii* (*Ld*NDT) with introduced cysteine pairs was created. Among them, *Ld*NDT_S104C_ (100% retained activity) was chosen as the most thermostable variant, displaying a six- and two-fold enhanced long-term stability when stored at 55 °C (t_1/2_^55^ ^°C^ ≈ 24 h) and 60 °C (t_1/2_^60^ ^°C^ ≈ 4 h), respectively. Moreover, the biochemical characterization revealed that *Ld*NDT_S104C_ showed >60% relative activity across a broad range of temperature (30–90 °C) and pH (5–7). Finally, to study the potential application of *Ld*NDT_S104C_ as an industrial catalyst, the enzymatic synthesis of nelarabine was successfully carried out under different substrate conditions (1:1 and 3:1) at different reaction times. Under these experimental conditions, the production of nelarabine was increased up to 2.8-fold (72% conversion) compared with wild-type *Ld*NDT.

## 1. Introduction

Nucleosides are essential molecules in many different biochemical pathways, such as nucleic acid synthesis, regulation and metabolism, and signal transduction. Consequently, nucleoside analogues (NAs) have attracted considerable attention as antiviral [1], anticancer [2], or antibacterial [3] active pharmaceutical ingredients (APIs) in the pharma industry.

With the growing environmental concern, nowadays, enzymatic synthesis of nucleoside APIs is gaining ground over the traditional chemical methodologies in the pharma industry [4,5,6]. Enzyme-mediated synthesis of NAs offers an ecofriendly, regio-, stereo-, and enantioselective alternative to traditional multistep and environmentally harmful chemical processes. To this effect, many different enzymes have been employed as practical catalysts for the mono and multi-enzymatic synthesis of NAs, including nucleoside phosphorylases [7,8], 2′-deoxyribosyltransferases [9,10], phosphoribosyltransferases [11,12], and nucleoside kinases [13,14,15,16] or deaminases [17,18,19], among others.

Despite the undeniable potential of biocatalysis as a valuable tool for the industrial production of NAs, several operational drawbacks hamper the application of enzymes as industrial biocatalysts [20,21]. Among these, achieving enzyme stabilization under harsh conditions used in industry is the most challenging from a practical standpoint. Modern biocatalysis demands new biocatalysts active and stable under aggressive reaction conditions, e.g., extreme pH values, high temperatures, or the presence of organic solvents and hazardous reagents. In this respect, the use of different strategies to get robust biocatalysts is a habitual practice in the pharma industry. These include the immobilization of the enzymes onto different matrixes [22,23], the use of enzymes from extremophilic organisms [24,25], or the tailor-made engineering of more stable enzymes [26,27].

The rational engineering of more stable variants is strongly dependent on structure-based computational design, which can identify hotspots on the protein sequence for mutagenesis to reach the optimization of flexible regions, the rigidification of the structure, or the introduction of specific stabilizing interactions and metal-chelating sites [28,29]. In this context, several computational tools are commonly used to predict stabilizing mutations, either based on sequence (consensus mutagenesis, proline substitutions, and disulfide bond formation) [30,31,32] or on structure knowledge (molecular dynamics, B-FITTER, constrain network analysis, and FoldX) [33,34,35,36].

**Figure 1:**
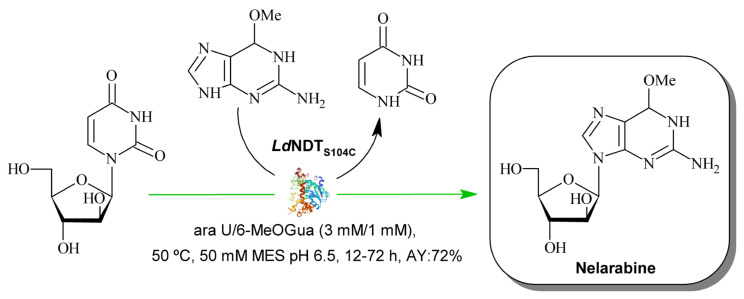
NDT-mediated synthesis of nelarabine. Ara U: arabinosyl uracil; MeOGua: 6-methoxyguanine; AY: assay yield.

Since *Ld*NDT displays a homohexameric assembly, similar to other reported bacterial homologs from *L. leichmannii, L. helveticus,* or *Bacillus psychrosaccharolyticus* [11,37,38], this oligomeric character opened up new design possibilities for the incorporation of intersubunit disulfide bridges by following a structure-guided approach. In this context, based on the in silico predictions, four variants (*Ld*NDT_V63C_, *Ld*NDT_V93C_, *Ld*NDT_S104C_, and the double mutant *Ld*NDT_V93C/S104C_) were produced and characterized. Among them, *Ld*NDT_S104C_ displayed similar activity to that shown by wild-type *Ld*NDT, but also an enhanced thermostability (up to six- and two-fold t_1/2_ enhancement when stored at 55 and 60 °C, respectively), which demonstrated the effect of this mutation on thermal stability. Finally, *Ld*NDT_S104C_ was successfully applied as a biocatalyst for the synthesis of nelarabine (Figure 1), improving the conversion displayed by the wild-type counterpart.

## 2. Results and Discussion

### 2.1. Computational Design of LdNDT Variants

The use of NDTs as biocatalysts for the synthesis of NAs has been extensively described [6,9,37,38,39,40,41,42,43,44,45]. However, even though NDT-mediated transglycosylation emerges as a versatile tool for industrial manufacturing of NAs, the long reaction times (24–48 h) required for the synthesis of some NAs (2′ and 3′ modified nucleosides) usually compromise the enzyme lifespan. Recently, we have described the presence of inter- and intra-subunit disulfide bridges (DSB) in NDT from *Desulfotalea psychrophila* (*Dp*NDT, PDB ID 7O62) [41], which contributes to stabilizing the protein folding. Inspired by these findings, we envisioned that the introduction of DSBs into *Lactobacillus delbrueckii* NDT structure may improve thermal stability.

**Figure 2:**
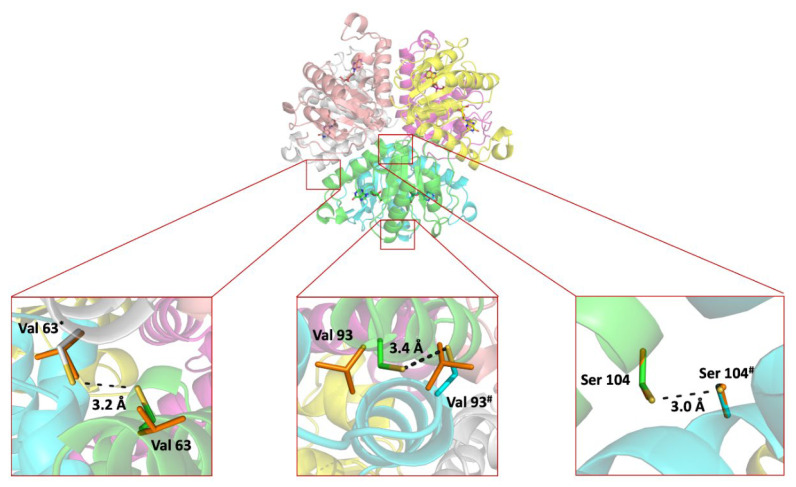
Overall cartoon representation of hexameric *Ld*NDT, including proposed mutations for the formation of inter- and intra-DSBs. The figure was prepared with PyMOL 2.5.

To this end, based on the high sequence identity (98%) with hexameric NDT from *L. leichmannii* (PDB ID 1F8Y) [46], a 3D model was constructed [47]. Based on the results of calculations with the SSBONDPredict [48] several inter- and intra-subunit DSBs were proposed. Moreover, the effect of these mutations on protein structure was analyzed using HotSpot Wizard [30]. Finally, four variants: *Ld*NDT_V63C_, *Ld*NDT_V93C_, *Ld*NDT_S104C_, and the double mutant *Ld*NDT_V93C/S104C_ were proposed as tentative candidates for in vitro experiments (Figure 2 and Table 1). 3D models for each *Ld*NDT variant were constructed, and MD simulations were run. Distances and angles for introduced Cys residues were consistent with disulfide bond formation [49,50]. 

The location of the disulfide bridges within the hexameric assembly of *Ld*NDT is indicated in the upper part of Figure 2, which shows *Ld*NDT along its three-fold molecular symmetry axis. It should be remarked here that introduction of just one cysteine in the amino acid sequence of the enzyme results in the introduction of three disulfide bridges in the assembly. Therefore, the global effects observed in thermal stability of the *Ld*NDT variants (see below) would result from a joint combination of the internal symmetry of the enzyme and the introduction of the Cys residue, namely, the effect of the mutation is amplified by the molecular symmetry. Conversely, the lower panels of Figure 2 show close-up views of the amino acid changes incorporated: V63C, V93C, and S104C (from left to right). The distances between sulfur atoms are within the range observed for disulfides, as well as those for the Cα-Cα distances (5.6 Å for V63C, 4.5 Å for V93C, and 6.5 Å for S104C; typical range: 3.0–7.5 Å).

### 2.2. Production of LdNDT Variants

Taking into account these previous features, the engineered *Ld*NDT variants were produced and purified (see Section 3). SDS-PAGE analysis of the purified variants revealed only one protein band with an apparent molecular mass of 18 kDa (Figure S1). Once the *Ld*NDT variants were purified, we assayed the *N*-2′-deoxyribosyltransferase activity in the wild-type and engineered variants under standard assay conditions (Table 1 and Figure S2).

As shown in Table 1, *Ld*NDT_V63C_ and *Ld*NDT_V93C/S104C_ variants exhibited a drastic loss of activity compared to the wild-type counterpart, whereas *Ld*NDT_V93C_ did not show a remarkable loss of activity under standard assay conditions. Interestingly, a similar activity was observed for the *Ld*NDT_S104C_ variant.

**Figure 3:**
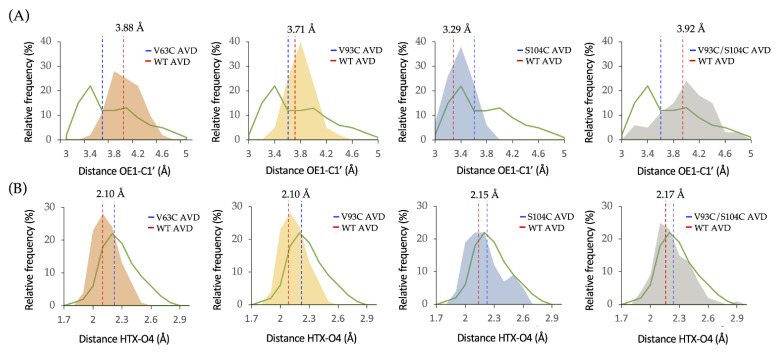
Overlap of distance distributions obtained in MD simulations of the different NDT-dUrd complexes. (**A**) d1 distance distributions for wild-type *Ld*NDT and mutants. (**B**) d2 distance distributions for wild-type *Ld*NDT and mutants. AVD: average distance; WT: *Ld*NDT; V63C: *Ld*NDT_V63C_; V93C: *Ld*NDT_V93C_; S104C: *Ld*NDT_S104C_; V93C/S104C: *Ld*NDT_V93C/S104C_.

In an attempt to explain the activity difference between *Ld*NDT variants, distances from OE1 of catalytic Glu98 to C1′ of dUrd (d1), as well as distances between H from the C-terminal (HTX) of catalytic Tyr157# and O4 of dUrd (d2), were measured (Figure 3). The evaluation of these distances through MD simulations may explain the distortion of the active site. As shown in Figure 3, the presence of cystine mutations leads to significant changes in the d1 distance (Figure 3A, average d1 distances) compared to the wild-type counterpart. However, no significant differences are observed in the d2 distance, and more importantly, the average values for all *Ld*NDT variants fall below 2.5 Å (Figure 3B, average d2 distances). To that effect, as previously reported, the productive complex is formed when d1 and d2 distances are less than 3.45 Å and 2.5 Å, respectively [51]. Interestingly, the MD simulation of *Ld*NDT_S104C_ reveals an average d1 distance located under 3.45 Å, which is consistent with the relative activity values reported in Table 1. For the rest of the mutants, the average d1 distance stands above 3.45 Å, which could be related to the observed lower activity values. More interestingly, the distribution curve for *Ld*NDT_S104C_ d1 distances falls within that shown for productive complex for *Ld*NDT (d1 < 3.45 Å) [51], which is not observed for the other variants. All in all, the computational analysis of enzyme-ligand complexes revealed that the presence of a cystine could distort the enzyme–ligand interactions and therefore interfere with substrate binding and catalysis. 

As a result of this first screening, strictly focused on enzymatic activity, variants with retained activities ≥ 85% were chosen as candidates for further experiments.

### 2.3. Thermal Stability

**Figure 4:**
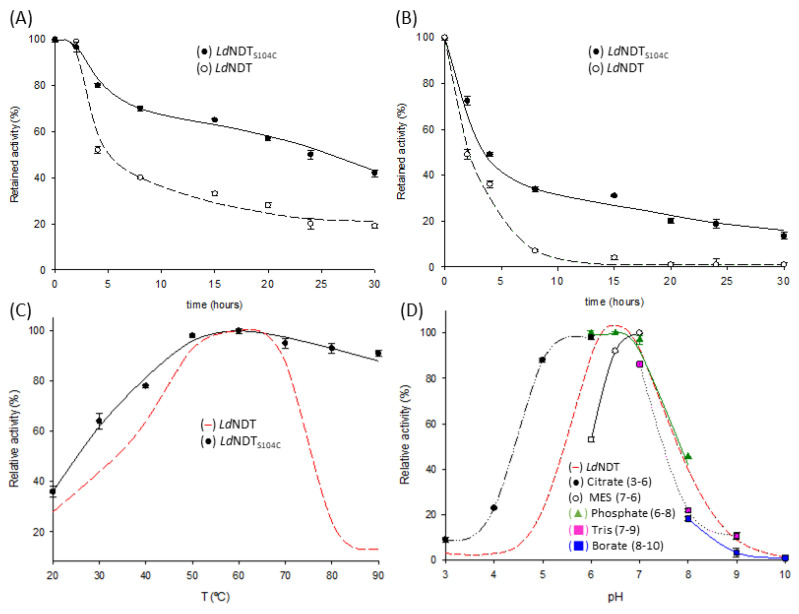
Thermal inactivation profile of *Ld*NDT and *Ld*NDT_S104C_ at different temperatures in 10 mM sodium phosphate, pH 7.0. (**A**) 55 °C. (**B**) 60 °C. (**C**) Temperature profile for *Ld*NDT_S104C_. (**D**) pH profile for *Ld*NDT_S104C_. All determinations were carried out in triplicate, and the standard error of the mean is calculated using the standard deviation.

The effect of temperature on the stability of *Ld*NDT, *Ld*NDT_V93C_, and *Ld*NDT_S104C_ was assayed. As shown in Figure 4, significant differences in thermal stability were observed for wild-type *Ld*NDT and mutant variants when incubated at 55 and 60 °C (Figure 4A,B).

Although *Ld*NDT losses ~50% of the initial activity in the first 4 h of incubation, almost no further drop in enzyme activity is observed until reaching 24 h of incubation at 55 °C (23% relative activity) (Figure 4A). *Ld*NDT_V93C_ displayed a similar tendency, losing ~50% of the initial activity in the first 4 h of incubation (t_1/2_^55 °C^ ≈ 4 h). However, the activity drops significantly in the following hours, observing 11% relative activity after 15 h of incubation (data not shown). In contrast, *Ld*NDT_S104C_ displays up to six-fold thermal stability enhancement (t_1/2_^55 °C^ ≈ 24 h) compared to the wild-type enzyme (Figure 4A). As a result of this first test, *Ld*NDT_S104C_ was selected for further thermostability experiments (60 °C) (Figure 4B). *Ld*NDT suffered a remarkable loss of activity at shorter incubation periods when stored at 60 °C (t_1/2_^60 °C^ ≈ 2 h), whereas a higher half-time was observed for *Ld*NDT_S104C_ (t_1/2_^60 °C^ ≈ 4 h, two-fold improvement). Additionally, *Ld*NDT and *Ld*NDT_S104C_ display similar long-term stability when stored at 55 °C in the presence of the reducing agent 1 mM DTT (Figure S3), probably due to the reduction of the disulfide bonds in *Ld*NDT_S104C_.

To deepen into the molecular basis of experimental results, MD simulations at different temperatures were carried out. Throughout these simulations, variations of RMSD (root mean square deviation of atomic positions); RMSF (root mean square fluctuation of protein backbone); and B-factor (so called the Debye-Waller factor, temperature factor, or atomic displacement parameter) values were evaluated as a measurement of the effect of mutation on the overall conformation and flexibility shown by the enzyme [52,53,54]. For that matter, higher variations in these values are associated with superior conformational changes and increased flexibility in the protein structure. Since disulfide bond formation leads to a more rigid structure, nonsignificant variations in the RMSD, RMSF, and B-factor values are expected when the temperature rises.

**Figure 5:**
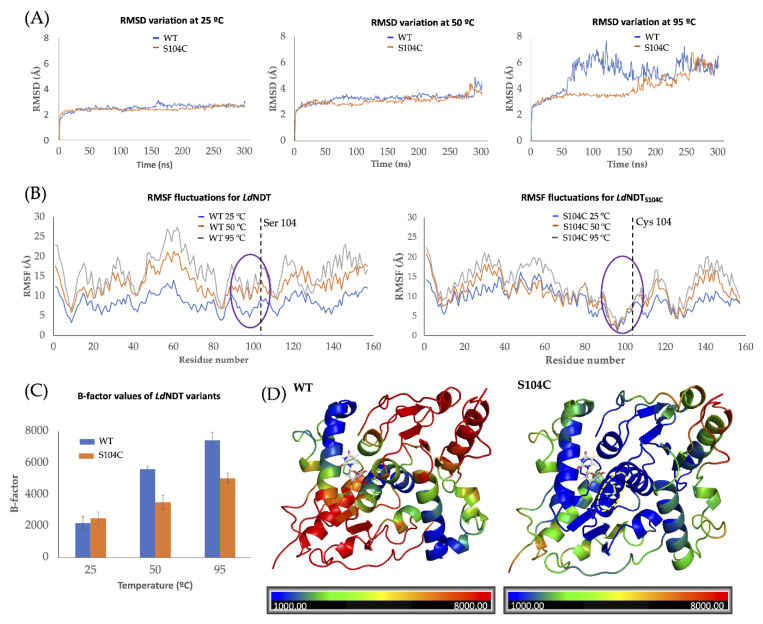
(**A**) RMSD variation of wild-type *Ld*NDT and *Ld*ND_S104C_ at 25 °C (left), 50 °C (center), and 95 °C (right). (**B**) Residue-by-residue RMSF fluctuations for *Ld*NDT (left) and *Ld*NDT_S104C_ (right) through MD simulations at different temperatures. A purple circle indicates amino acids present in the vicinity of catalytic Glu98, while the dashed line indicates the 104 positions. (**C**) Average B-factor values for *Ld*NDT and *Ld*NDT_S104C_ at different temperatures. (**D**) Cartoon representation of *Ld*NDT (left) and *Ld*NDT_S104C_ (right) colored according to the B-factor values calculated at 95 °C.

As shown in Figure 5A, similar RMSD values are observed for both *Ld*NDT and *Ld*NDT_S104C_ through MD simulations in the 25–50 °C temperature range. When simulations were performed at 95 °C, the RMSD values significantly increase for *Ld*NDT after 50 ns, while a similar tendency is observed for *Ld*NDT_S104C_ after 150 ns. Furthermore, to explain these RMSD differences, the RMSF variations were also evaluated for both enzymes at different temperatures (Figure 5B). Attending to the obtained results, a significant decrease in RMSF values was observed in the vicinity of the Cys104 position for *Ld*NDT_S104C_, probably related to a structural rigidification by the presence of a disulfide bond. In addition, MD simulations of *Ld*NDT_S104C_ show lower RMSF values for catalytic residues Glu98 and Tyr157# at higher temperatures (compared to wild-type *Ld*NDT), which is consistent with the long-term operational stability observed for *Ld*NDT_S104C_.

Additionally, the overall flexibility of enzymes was analyzed by the measurement of average B-factor values through independent MD simulations at different temperatures (25–95 °C). The computational results revealed a 1.4-fold increment of the B-factor average value for *Ld*NDT_S104C_ and a 2.5-fold increase for *Ld*NDT (Figure 5C) when the temperature rises from 25 °C to 50 °C. Moreover, broader differences were observed in the 25–95 °C range, displaying 2.0- and 3.3-fold increments for *Ld*NDT_S104C_ and *Ld*NDT, respectively. Finally, attending to their B-factor cartoon representation, significant differences are detected in the flexibility of *Ld*NDT and *Ld*NDT_S104C_ (Figure 5D).

### 2.4. Biochemical Characterization

Taking into account the abovementioned results, *Ld*NDT_S104C_ emerges as the most valuable candidate from an industrial perspective. Therefore, the next step was to establish the optimal conditions to reach maximum activity. To this end, the effect of temperature and pH on *Ld*NDT_S104C_ activity was studied (Figure 4C,D) and compared to those results previously reported for *Ld*NDT [11]. When compared to its wild-type counterpart, *Ld*NDT_S104C_ displayed high activity values (more than 60%) across a broader temperature range (from 30 °C to 90 °C) (Figure 4C). It is particularly interesting to note that a negligible loss of activity was observed at 80 °C and 90 °C for the mutant enzyme, while just a ~20% relative activity was reported for wild-type *Ld*NDT at the same temperatures [11]. Thus, besides the enhanced thermal stability, the proposed mutation could also improve the enzyme activity at extremely high temperatures. As reported for *Ld*NDT, the maximum activity for *Ld*NDT_S104C_ was observed at 60 °C. Regarding the pH dependence, *Ld*NDT_S104C_ displays a similar tendency to that shown by *Ld*NDT [11] (Figure 4D), with a maximum in the pH range of 6–7.

### 2.5. Enzymatic Synthesis of Nelarabine

Nelarabine, a water-soluble prodrug of ara G licensed by SmithKline Beecham in 2005, received approval for the treatment of T-cell acute lymphoblastic leukemia [1]. Chemical methods for nelarabine synthesis involved condensation between an arabinosyl derivative and 6-methoxy guanosine, which usually leads to anomeric mixtures and, therefore, to a significant decrease in the efficiency of the process [55]. In addition, these strategies usually employ expensive and environmentally unfriendly chemical reagents and organic solvents. Therefore, the underlying need for waste reduction and high (enantio)-selectivities fostered the introduction of biocatalysis as a sustainable technology for the industrial synthesis of nelarabine.

In contrast, enzymatic transglycosylation emerges as a cost-effective and eco-friendly alternative to chemical methods. In recent years, nucleoside phosphorylases (NPs) have been widely employed as powerful catalysts for nelarabine synthesis [6,56]. However, the high cost of α-D-arabinofuranose 1-phosphate (sugar donor) is a serious constraint for their industrial application. In this context, different alternatives are now under study by pharma industries. Among them, the NDT-mediated synthesis of nelarabine from arabinosyl nucleosides and 6-methoxyguanine (6-MeOGua) could offer an eco-friendly but also cheaper process alternative [10,39,57,58,59,60].

Herein, the authors propose an NDT-mediated one-pot synthesis of nelarabine from ara U and 6-MeOGua. Since long reaction times are required for the NDT-mediated synthesis of arabinosyl nucleosides [10,39,57,58,59,60], it is expected that a more stable NDT (*Ld*NDT_S104C_) would allow an increase in conversion rates.

**Figure 6:**
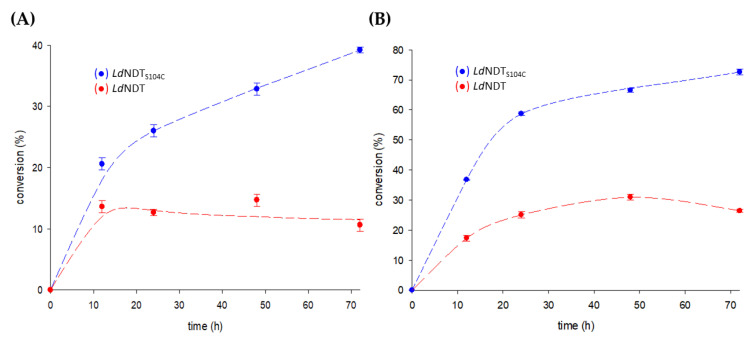
Time–course of enzymatic production of nelarabine catalyzed by *Ld*NDT and *Ld*NDT_S104C_. (**A**) ara U:6-MeOGua (1:1). (**B**) ara U:6-MeOGua (3:1). All determinations were carried out in triplicate, and the standard error of the mean is calculated using the standard deviation.

As shown in Figure 6A, the variant *Ld*NDT_S104C_ displayed around a 3.7-fold higher production yield (compared to wild-type *Ld*NDT) when using a 1:1 nucleoside/base ratio. The observed improvement could be due to the enhanced thermostability displayed by *Ld*NDT_S104C_, which allows the enzyme to be more active during the long reaction periods employed for the production of nelarabine. Furthermore, to improve the nelarabine production yields, a 3:1 substrate ratio was employed under the same reaction conditions (Figure 6B). Interestingly, the conversion rates were significantly improved for both *Ld*NDT (from 10 to 26%) and *Ld*NDT_S104C_ (from 39 to 72%). Compared to previous results (1:1 ratio experiments), the differences in the conversion rates for both enzymes were diminished; however, a 2.8-fold increment was still observed for *Ld*NDT_S104C_. Once again, when compared to *Ld*NDT, the observed improvement could be due to the enhanced thermostability displayed by *Ld*NDT_S104C_, which allows the enzyme to be more active during the long reaction periods.

**Figure 7:**
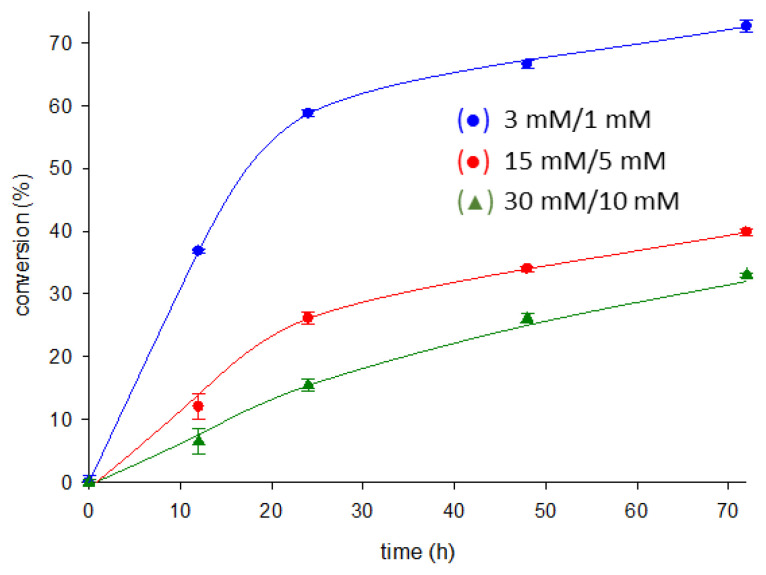
Time–course of the enzymatic production of nelarabine catalyzed by *Ld*NDT_S104C_ at different substrate concentrations: ara U (3–30 mM) and 6-MeOGua (1–10 mM). All determinations were carried out in triplicate, and the standard error of the mean is calculated using the standard deviation.

Finally, as a proof of concept of the potential of *Ld*NDT_S104C_ as an industrial biocatalyst, we performed the synthesis of nelarabine at high concentrations of ara U (15–30 mM) and 6-MeOGua (5–10 mM) over different reaction times (ranging from 12 to 72 h) at 50 °C since thermal stability of the enzyme was guaranteed (Figure 7).

Based on the previous literature for wild-type *Ld*NDT [11], high promiscuity on nucleobase recognition is expected. Together with the enhanced thermostability displayed by *Ld*NDT_S104C_, suggest that other different APIs could be synthesized through this methodology, such as vidarabine (adenine arabinoside, ara A), fludarabine (2-fluoroadenine arabinoside, ara FA), or clofarabine (2-chloradenine-2′-fluoroarabinoside), among others. However, despite this enzymatic approach vastly surpassing chemical procedures in terms of efficiency (one-pot regio- and enantioselective reactions) or sustainability (green conditions and aqueous buffers), from an industrial perspective, several operational constraints need to be addressed. One of the major drawbacks is the high cost of production and purification of recombinant enzymes, which makes necessary the capability of reusing the catalyst on successive reactions to reach a cost-effective process. In this regard, enzyme immobilization offers valuable strategies to overcome this operational handicap but also the possibility to integrate the immobilized catalyst in continuous flow reactors.

## 3. Materials and Methods

### 3.1. Materials

All the substrates and reagents for microbiology experiments were from Condalabs (Madrid, Spain). Moreover, HPLC solvents and buffers were purchased from Sigma-Aldrich (Madrid, Spain). All other compounds involved in enzyme reactions were from Carbosynth Ltd. (Compton, UK).

### 3.2. Gene Expression and Protein Purification

All the DNA constructions were purchased from Genscript (Piscataway, NJ, United States) as *Nde*I-*Eco*RI fragments subcloned into the expression vector pET22b(+). The recombinant plasmids were used to transform *E. coli* BL21 (DE3) cells under conditions described elsewhere [11]. The overexpression of wild-type *Lactobacillus delbrueckii* NDT (UniProtKB Q1GC13) and variants and the cell extract preparation for protein purification were performed following the previous protocol reported by Acosta et al. [11].

*Ld*NDT and mutant variants were purified by ammonium sulfate precipitation. To this end, the supernatant obtained after centrifugation of the lysed cells was brought to 40% (NH_4_)_2_SO_4_ saturation for the selective precipitation of the enzymes. The prepared solutions were incubated for 1 h at 4 °C and then centrifugated at 16,500× *g*. The collected pellets were resuspended in 10 mM sodium phosphate buffer, pH 7.0, heated at 67 °C for 7 min, and further incubated at 4 °C. After a centrifugation step (15,800× *g*), the supernatant was collected. Then, the protein mixture was purified by size-exclusion chromatography (HiLoad 16/60 Superdex 200, GE Healthcare, Madrid, Spain) (50 mM sodium phosphate, pH 7.0). The fractions comprising pure enzymes were identified by an SDS-PAGE and further confirmed by an activity assay. Finally, the protein concentration was spectrophotometrically quantified as described before [11].

### 3.3. N-2′-Deoxyribosyltransferase Activity Assay

The standard assay was carried out using 0.3 μg of the pure enzyme with 10 mM substrates (2′-deoxyuridine, dUrd, and 10 mM adenine, Ade) in 50 mM MES buffer, pH 6.5 (reaction volume 40 μL) at 50 °C for 5 min (300 rpm). Then, the reaction was stopped, and the samples were half-diluted with water for HPLC analysis. One unit of activity (U) was established as the milligrams of the enzyme that produces 1 μmol of product per min (IU).

### 3.4. Thermal Inactivation

*Ld*NDT and mutants were stored at different temperature conditions (4 and −80 °C) for around 200 days. Moreover, to determine the effect of temperature on the operational stability of *Ld*NDT, *Ld*NDT_V93C_, and *Ld*NDT_S104C_, 0.3 µg of purified enzyme were incubated in 10 mM sodium phosphate buffer, pH 7.0, for 30 h at different temperatures (55–60 °C). During these periods, the enzymatic activity was systematically assayed. Moreover, *Ld*NDT and *Ld*NDT_S104C_ were also incubated in the presence of 1 mM DTT (dithiothreitol) for 24 h.

### 3.5. Biochemical Characterization

To establish the optimal operational conditions, the effect of pH and temperature on enzyme activity was assayed. The influence of pH on *Ld*NDT_S104C_ activity was tested using different reaction buffers (50 mM sodium citrate, pH 3–6; 50 mM MES, pH 6–7; 50 mM Tris-HCl, pH 7–9; 50 mM sodium phosphate, pH 6–8; and 50 mM sodium borate, pH 8–10). Similarly, the effect of temperature on *Ld*NDT_S104C_ activity was assayed across the 20–90 ºC range.

### 3.6. Enzymatic Synthesis of Nelarabine

To evaluate the potential use of *Ld*NDT_S104C_ as a catalyst, the enzymatic production of nelarabine was carried out under different reaction conditions (reaction time and substrate ratio). To this end, 10 μg of *Ld*NDT or *Ld*NDT_S104C_ were incubated with 1 mM arabinosyl uracil (ara U) and 6-methoxyguanine (6-MeOGua) in 50 mM MES buffer pH 6.5, at 50 °C in a final volume of 40 μL, with 300 rpm orbital shaking at different reaction times (12–72 h). Thereafter, the reactions were stopped following the standard assay described above. Further experiments were carried out at a 3:1 nucleoside/base substrate ratio (ara U/6-MeOGua; 3 mM/1 mM) to improve the conversion yield. The enzymatic activity was quantified by HPLC as described above.

Finally, to demonstrate the industrial applicability of *Ld*NDT_S104C_, the enzymatic synthesis of nelarabine from high concentrations of ara U (15–30 mM) and 6-MeoGua (5–10 mM) was performed.

### 3.7. Molecular Modeling

The hexameric model for apo *Ld*NDT was constructed based on the structure of type II 2′-deoxyribosyltransferase from *L. leichmannii* complexed with 5-methyl-2′-deoxypseudouridine (PDB ID 1F8Y) (98% sequence identity). Titratable residues were properly protonated at pH 6.5 using the H^++^ 3.0 webserver [61]. Then, dUrd was manually docked into the active site through structural best-fit superposition onto the former ligand (5-methyl-2′-deoxypseudouridine). Previously, the Antechamber (AmberTools16) [62] program was utilized for the optimization of the ground state geometry of the dUrd molecule, as well as for computing its electrostatic potential using quantum mechanical HF/6-31G* wavefunction fitted to the atoms as RESP charges.

The consistency of our 3D model was validated through MD simulation using the leaprcff14SB force field. The MD simulation was run in the Single-Precision-Fixed-Precision (SPFP) mode implemented in the pmemd.cuda module of Amber16. The *Ld*NDT:dUrd complex was simulated as a hexamer, and the complex was embedded in a cubic box of TIP3P water molecules [63], including 27 Na^+^ ions, to achieve charge neutrality. As described before for this enzyme [47], the total energy of the system was minimized in three consecutive steps (3 × 20,000 cycles). In the first 5000 cycles, a steepest descendent method was employed, while the following minimization cycles were calculated by the conjugate gradient method. After a 200 ps heating (100–300 K) restricting the movement of all solute atoms [64], the simulation was run with a fixed volume (NVT ensemble) as previously described [47].

Once the consistency of the model was confirmed, the system was employed for the construction *Ld*NDT variants to improve the thermostability. Those mutations were predicted based on both structural and sequence information [41,51,65]. Mutations leading to disulfide bond formation were predicted based on the structural knowledge using the Neural Network Model AI algorithm implemented in SSBONDPredict software [48]. Those mutations were further confirmed by the sequence analysis through the HotSpot Wizard server [30]. All in all, three potential mutable positions were identified and evaluated by MD simulations following the abovementioned protocol. In this respect, selected amino acids were mutated to cysteine residues with PyMOL [66] for the construction of four *Ld*NDT variants: *Ld*NDT_V63C_, *Ld*NDT_V93C_, *Ld*NDT_S104C_, and *Ld*NDT_V93C/S104C_. Cystines were formed by tleap software available in AmberTools16, and the suitableness of the models was confirmed by MD simulations. Results for the MD simulations were analyzed with the cpptraj module available in AmberTools16 [62].

### 3.8. QM/MM MD Simulations

MD simulations for all mutant candidates were run as described before to refine the *Ld*NDT:dUrd Michaelis complex for the first half-reaction. The distances between the reactant OE1 of Glu98 and the C1’ anomeric carbon of dUrd, as well as H from the C-terminal of Tyr157# and O4 of dUrd, were monitored. The best snapshot was selected as previously described by Del Arco and co-workers [65] and further simulated for 50 ps through QM/MM MD simulation without any restraint. For the first half-reaction, the ligand (dUrd) and catalytic residues, Glu98 and Tyr157#, were included in the QM region. As previously described [65], the QM region was treated at the self-consistent-charge density-functional tight-binding method (SCC-DFTB) [67] using the DFTB3 formulation, whereas the rest of the system was treated with MM MD, as described above. The results for QM/MM MD simulations were analyzed with the cpptraj module available in AmberTools16 [62].

### 3.9. MD Simulations for Thermal Stability Predictions

Once the best mutant candidate was selected (*Ld*NDT_S104C_), MD simulations at different temperatures were run to compare the thermal stabilities of *Ld*NDT and *Ld*NDT_S104C_. More specifically, these simulations give an insight into the flexibility of the enzymes at high temperatures, which can be related to thermal stability [52]. The flexibility was evaluated through the measurements of the RMSD, RMSF, and B-factor values [52,53,54] through the MD simulations. In this sense, these simulations were made following the above-described protocols but at different temperatures ranging from 300 K to 400 K. the RMSD, RMSF, and B-factor values from the MD simulations were analyzed with the cpptraj module available in AmberTools16 [62]. The MD simulations were carried out in triplicate.

### 3.10. Analytical Methods

The quantitative determination of the products was performed by HPLC analysis using an ACE 5-μm C18-PFP 250 mm × 46 mm column (Avantor-ACE) under the following conditions: (i) discontinuous gradient (100–90% triethylammonium acetate and 0–10% acetonitrile) for 15 min and (ii) an isocratic elution (90% triethylammonium acetate and 10% acetonitrile) for 7 min. The retention times for the substrates and products were outlined hereafter: 6-methoxyguanine (6-MeOGua): 7.72 min, adenine (Ade): 10.01 min, uracil (Ura): 4.68 min, nelarabine: 15.30 min, arabinosyl uracil (ara U): 13.52 min, 2′-deoxyuridine (dUrd): 8.31 min, and 2′-deoxyadenosine (dAdo): 13.93 min.

## 4. Conclusions

Herein, we report the structure-guided design of a novel thermostable NDT based on the DSB design in *Ld*NDT. After a first screening focused on enzyme activity, *Ld*NDT_V93C_ and *Ld*NDT_S104C_ were selected as candidates for a second screening strictly focused on operational stability. Since the experimental results demonstrated *Ld*NDT_S104C_ as the most stable catalyst, this variant was successfully employed as a catalyst in the enzyme production of nucleoside analogs at long reaction times. Interestingly, a remarkable effect on nelarabine production (2.8-fold increment, 72% conversion) was observed when compared with that shown for *Ld*NDT, which reinforces our initial hypothesis.

## Figures and Tables

**Table 1:** NDT-mediated synthesis of 2′-deoxyadenosine (dAdo) from 2′-deoxyuridine (dUrd) and adenine (Ade) ^a^.

Enzyme	Disulfide Bridge Localization	Specific Activity (IU mg^−1^_enz_)	Relative Activity (%)
*Ld*NDT	-	191.83 ± 1.3	96
*Ld*NDT_V63C_	Interdimer	117.01 ± 3.0	59
*Ld*NDT_V93C_	Intradimer	176.37 ± 2.4	88
*Ld*NDT_S104C_	Intradimer	192.03 ± 4.0	100
*Ld*NDT_V93C/S104C_	Intradimer	106.88 ± 1.8	54

^a^ Reaction conditions: 0.3 μg of pure enzyme in 40 μL at 50 °C, 5 min. (Substrates) = 10 mM, 50 mM MES, pH 6.5. All determinations were carried out in triplicate, and the standard error of the mean is calculated using the standard deviation.

## Data Availability

Not applicable.

## Supplementary Materials

The following supporting information can be downloaded at https://www.mdpi.com/article/10.3390/ijms231911806/s1.
