# Supplementary material for: Rational Design of a Thermostable 2′-Deoxyribosyltransferase for Nelarabine Production by Prediction of Disulfide Bond Engineering Sites
Source: Int J Mol Sci. 2022 Oct 5;23(19):11806. doi: 10.3390/ijms231911806 (PMC9570332; doi:10.3390/ijms231911806)
Supplement: Supplementary file 1 [file ijms-23-11806-s001.zip › ijms-1874961-supplementary.pdf]

International Journal of *Molecular Sciences*

MDPI

Article

Rational design of a thermostable 2′-deoxyribosyltransferase for nelarabine production by prediction of disulfide bond engineering sites

Guillermo Cruz [1], Javier Acosta [1], Jose Miguel Mancheño [2], Jon Del Arco [1,] and Jesús Fernández-Lucas [1,*]

[1] Applied Biotechnology Group, Universidad Europea de Madrid, Urbanización El Bosque, Calle Tajo, s/n, 28670 Villaviciosa de Odón (Madrid), Spain. guille.sle@icloud.com (G. C), jacosta19.ja97@gmail.com (J. A.), jon.delarco@universidadeuropea.es (J. D.)

[2] Department of Crystallography and Structural Biology, Institute Rocasolano (CSIC), Serrano 119, 28006 Madrid, Spain, jm.mancheno@csic.es (J. M.)

*Correspondence: jesus.fernandez2@universidadeuropea.es (J. F.)

Electronic Supplementary Information

Figures

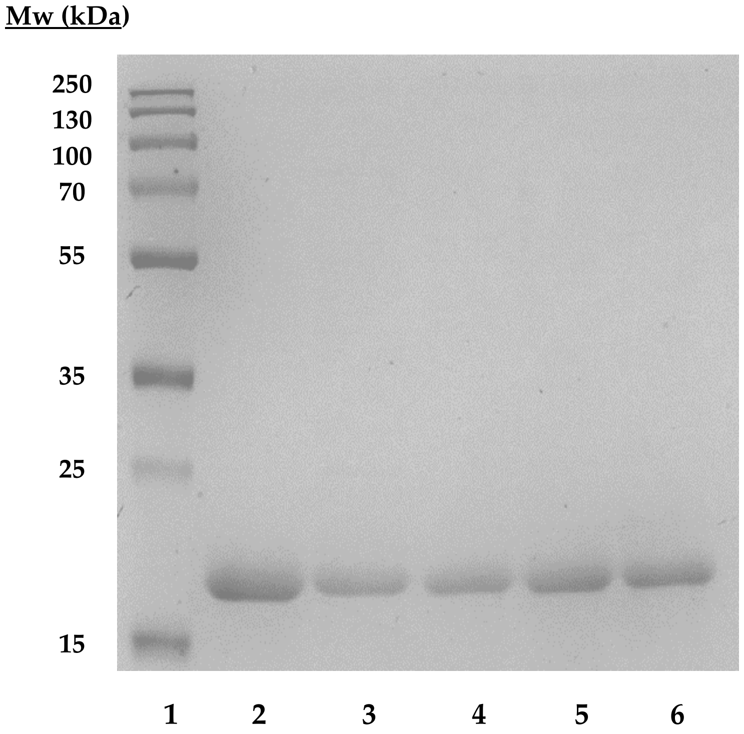

Figure S1. SDS-PAGE analysis of pure *Ld*NDTwt and mutant enzyme samples. **Lane 1.** Prestained standard proteins (Thermoscientific used as molecular weight markers. **Lane 2.** Pure fraction of *Ld*NDTwt. **Lane 3.** Pure fraction of *Ld*NDT$_{V64C}$. **Lane 4.** Pure fraction of *Ld*NDT$_{V93C}$. **Lane 5.** Pure fraction of *Ld*NDTS$_{104C}$. **Lane 6.** Pure fraction of *Ld*NDT$_{V93C/S104C}$. *Ld*NDT: NDT from *Lactobacillus delbrueckii*.

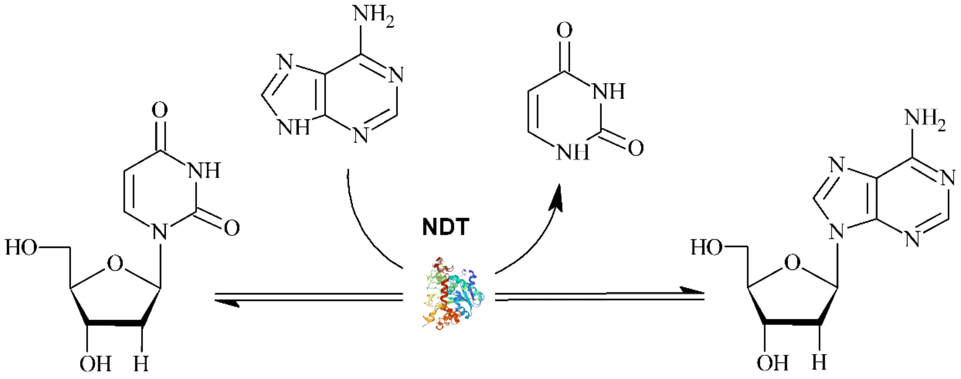

Figure S2. Enzymatic synthesis of 2′-deoxyadenosine (dAdo) from 2′-deoxyuridine (dUrd) and adenine catalyzed by 2′-deoxyribosyltransferases (NDTs).

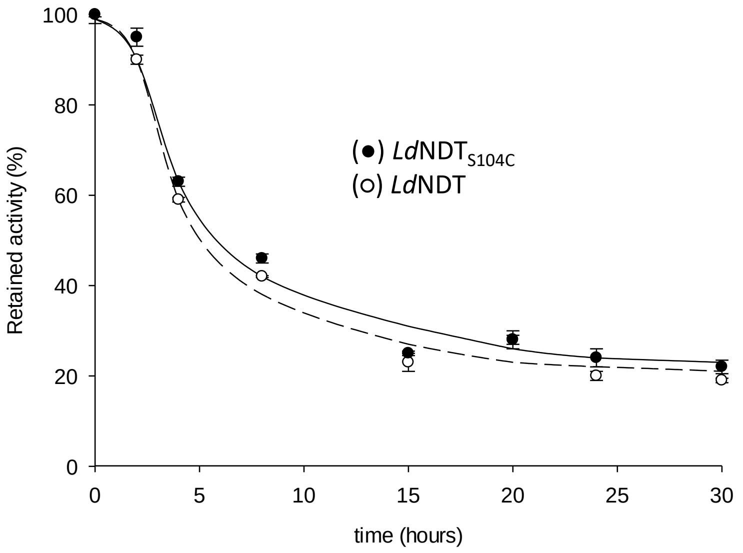

Figure S3. Thermal inactivation profile of *Ld*NDT and *Ld*NDT$_{S104C}$ at 55 ºC in 10 mM sodium phosphate, pH 7.0, in the presence of 1 mM DTT. All determinations were carried out in triplicate and the standard error of the mean is calculated using the standard deviation.
